# In Vivo Confocal Microscopy Cellular Features of Host and Organism in Bacterial, Fungal, and *Acanthamoeba* Keratitis

**DOI:** 10.1016/j.ajo.2018.03.010

**Published:** 2018-06

**Authors:** Jaya D. Chidambaram, Namperumalsamy V. Prajna, Srikanthi Palepu, Shruti Lanjewar, Manisha Shah, Shanmugam Elakkiya, David Macleod, Prajna Lalitha, Matthew J. Burton

**Affiliations:** a International Centre for Eye Health, London School of Hygiene & Tropical Medicine, London, United Kingdom; b Aravind Eye Hospital, Madurai, India; c Aravind Medical Research Foundation, Madurai, India; d Cornea Department, Moorfields Eye Hospital, London, United Kingdom

## Abstract

**Purpose:**

To determine cellular features of fungal (FK), *Acanthamoeba* (AK), and bacterial keratitis (BK) using HRT3 in vivo confocal microscopy (IVCM).

**Design:**

Prospective observational cross-sectional study.

**Methods:**

Eligible participants were adults with microbiologically positive FK, AK, or BK, of size ≥ 3 mm, attending Aravind Eye Hospital from February 2012 to February 2013. Exclusion criteria were descemetocele or perforation. At presentation, IVCM imaging was performed, then corneal scrapes were obtained for culture/light microscopy. An experienced grader (masked to microbiology/clinical features) assessed IVCM images for presence/absence of normal keratocyte-like morphology, stellate interconnected cells with/without visible nuclei, dendritiform cells (DFCs), inflammatory cells in a honeycomb distribution, and organism features. Statistical significance was assessed by logistic regression, adjusted for age, sex, ulcer size, and symptom duration. Main outcome measures were presence/absence of IVCM features in FK, AK, BK.

**Results:**

A total of 183 participants had FK, 18 AK, 17 BK. *Acanthamoeba* appeared as bright spots (16/18, 89%), double-walled cysts (15/18, 83%), or signet rings (3/18, 17%), and often formed clusters after topical steroid use (univariable odds ratio [OR] 9.98, 95% confidence interval [CI] 1.02-97.96, *P* = .048). BK was associated with bullae in anterior stroma (OR 9.99, 95% CI: 3.11-32.06, *P* < .001). Honeycomb distribution of anterior stromal inflammatory cells was associated with FK (univariable OR 2.74, 95% CI: 1.01-7.40, *P* = .047). *Aspergillus* ulcers were associated with stromal DFCs (OR 11.05, 95% CI: 1.49-82.13, *P* = .019) and *Fusarium* ulcers with stellate appearance of interconnected cell processes with nuclei (OR 0.24, 95% CI: 0.09-0.65, *P* = .005).

**Conclusion:**

Specific cellular and structural features observed using IVCM in microbial keratitis may be associated with organism.

In vivo confocal microscopy (IVCM) has been found to be a useful aid in the detection of organisms such as *Acanthamoeba* and fungi in human microbial keratitis (MK).^1^ Laser-scanning HRT3 IVCM enables higher-resolution imaging compared to white light IVCM (eg, Confoscan), thus allowing better visualization of cellular changes during healing in the cornea.^2^ Normal keratocytes have a specific appearance in IVCM images, with bright ovoid nuclei and barely visible cellular processes.^2^ After injury, keratocytes differentiate into fibroblasts and then myofibroblasts,^3^ and reduce their production of molecules that contribute to cellular transparency (eg, corneal crystallins), allowing greater visibility of their cellular processes with IVCM.^4^, ^5^ It has also been postulated that cellular changes associated with apoptosis may be visualized with IVCM, with absence of visible nuclei within cells and a granular appearance of intracellular contents.^2^ Presence of “spindle-like” opacities within cells in IVCM images of the cornea may represent intracellular actin or microtubules associated with myofibroblasts, as observed in immunohistochemical studies of these cell types.^6^ Additional cellular changes that have been noted using IVCM in MK are an increase in dendritiform cells (DFCs) (which become enlarged with long processes compared to their appearance in the normal cornea)^7^ and an influx of inflammatory cells.^8^ After corneal abrasion in a mouse model, HRT3 IVCM live imaging has shown that the initial inflammatory cells entering the stroma after injury migrate preferentially along keratocyte cellular processes and these processes are interconnected in a stellate, honeycomb-like pattern.^9^

Although many studies have looked at the diagnostic accuracy of IVCM for identifying a pathogen,^1^, ^10^, ^11^, ^12^ few have looked at the cellular changes in the cornea observed using IVCM in human MK.^7^, ^8^, ^13^ Since the molecular changes that occur in human MK caused by different organisms are subtly different,^14^ we postulated that the cellular changes in the cornea during infection may be different enough to allow use of these features to predict the infecting organism, even when the organism itself may not be apparent in the scan. In a prospective cohort of patients with moderate-to-severe bacterial keratitis (BK), *Acanthamoeba* keratitis (AK), and fungal keratitis (FK) in South India, we determined the IVCM appearance of the cornea at the cellular level. In addition, we documented specific features of organisms themselves that could be detected with this imaging modality, such as the “bright spot,” “double-walled cyst,” or “signet ring” appearance of *Acanthamoeba* cysts^15^, ^16^; spore-like structures along fungal filaments,^17^ or fine-beaded filamentous appearance of *Nocardia* sp.^18^, ^19^

## Methods

This study was prospectively approved by the Ethics Committees of the Indian Council for Medical Research, Aravind Eye Hospital, Tamil Nadu, India, and the London School of Hygiene and Tropical Medicine. As previously described, all patients gave written informed consent before enrolment; illiterate participants gave informed consent with a witnessed thumbprint on the study consent form (as approved by the above Ethics Committees).^1^ The tenets of the Declaration of Helsinki were adhered to during conduct of this study.

From February 28, 2012 to February 28, 2013, consecutive patients presenting to the Cornea Clinic of Aravind Eye Hospital, Madurai, Tamil Nadu, India were assessed for eligibility with the following inclusion criteria: age ≥ 18 years, stromal infiltrate diameter ≥ 3 mm, presence of overlying epithelial defect, and signs of acute inflammation. Patients were excluded if the ulcer had a descemetocele or >80% corneal thinning as assessed by slit-lamp examination (since applanation for IVCM could not safely be done in these patients), prior history of herpetic keratitis, or Snellen visual acuity worse than 6/60 in the unaffected eye, or if microbiologically negative (ie, culture and light microscopy) and IVCM-negative for any organism. At enrollment, data from a focused clinical history and slit-lamp examination were recorded. The cornea specialist examined every study participant and management was as per standard of care for microbial keratitis at Aravind Eye Hospital.

### In Vivo Confocal Microscopy Imaging

IVCM imaging of the corneal ulcer was performed using the HRT3 with Rostock Corneal Module (Heidelberg Engineering, Heidelberg, Germany) immediately prior to corneal scraping for microbiological tests, as described in detail elsewhere.^10^ Proparacaine 0.5% eye drop anesthesia was used (Aurocaine; Aurolab, Madurai, India) and the Rostock corneal module (Heidelberg Engineering) with 63× objective lens (Nikon, Tokyo, Japan) was gently applanated to the corneal surface. The HRT3 IVCM was used in volume scan mode, which consists of a z-stack of 40 images covering a total of 80 μm corneal depth, each image with optical slice thickness of 2 μm. Images were obtained at the center of the ulcer and ulcer margins (12-, 3-, 6-, and 9-o'clock positions) with repeated volume scans performed to image the full depth of the cornea at each location, where possible.

After IVCM imaging, corneal scrapes were obtained from the leading margin and base of the ulcer to identify the causative organism via culture and light microscopy, using standard procedures described in detail elsewhere.^1^ For culture- and light microscopy–negative ulcers, 5 experienced IVCM graders assessed the IVCM images to determine the presence/absence of fungal hyphae or *Acanthamoeba* cysts, as described in our previous report.^1^

### In Vivo Confocal Microscopy Grading

**Figure 1:**
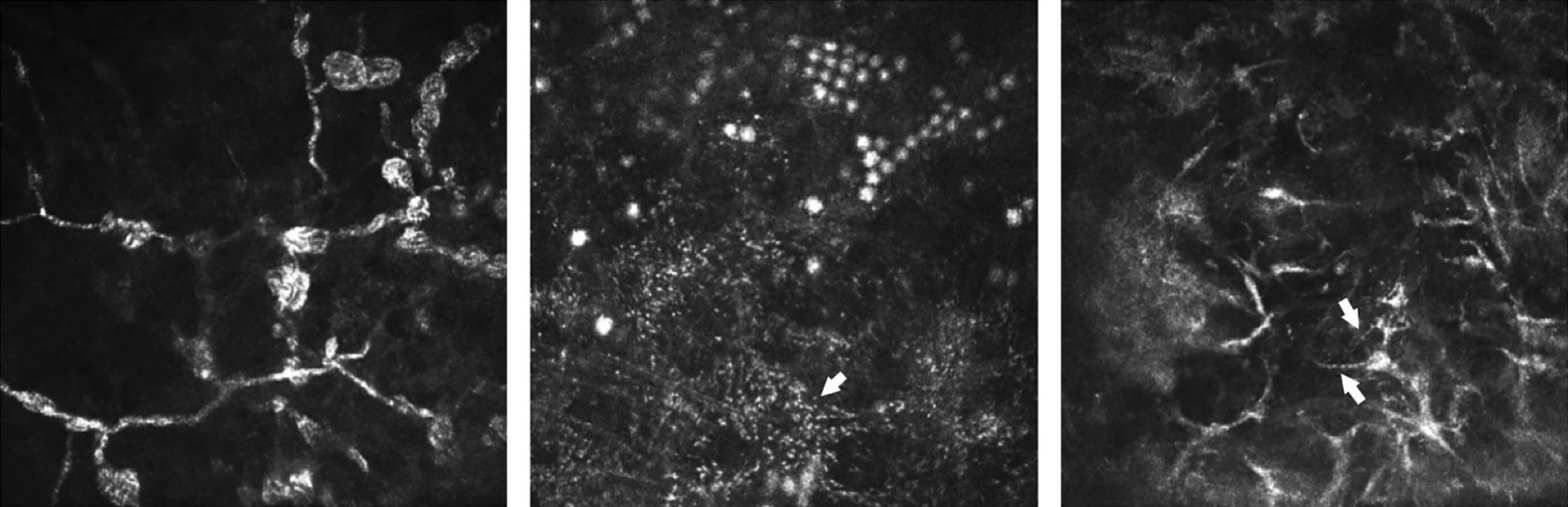
In vivo confocal microscopy (IVCM) images showing: (Left) spore-like structures along fungal filaments; (Middle) *Acanthamoeba* cysts forming lines and clusters (with presence of granules within the stellate interconnected cellular processes shown by arrow); and (Right) fine beaded filamentous appearance of *Nocardia* sp. (indicated by arrows). Each IVCM image measures 400 × 400 μm.

**Figure 2:**
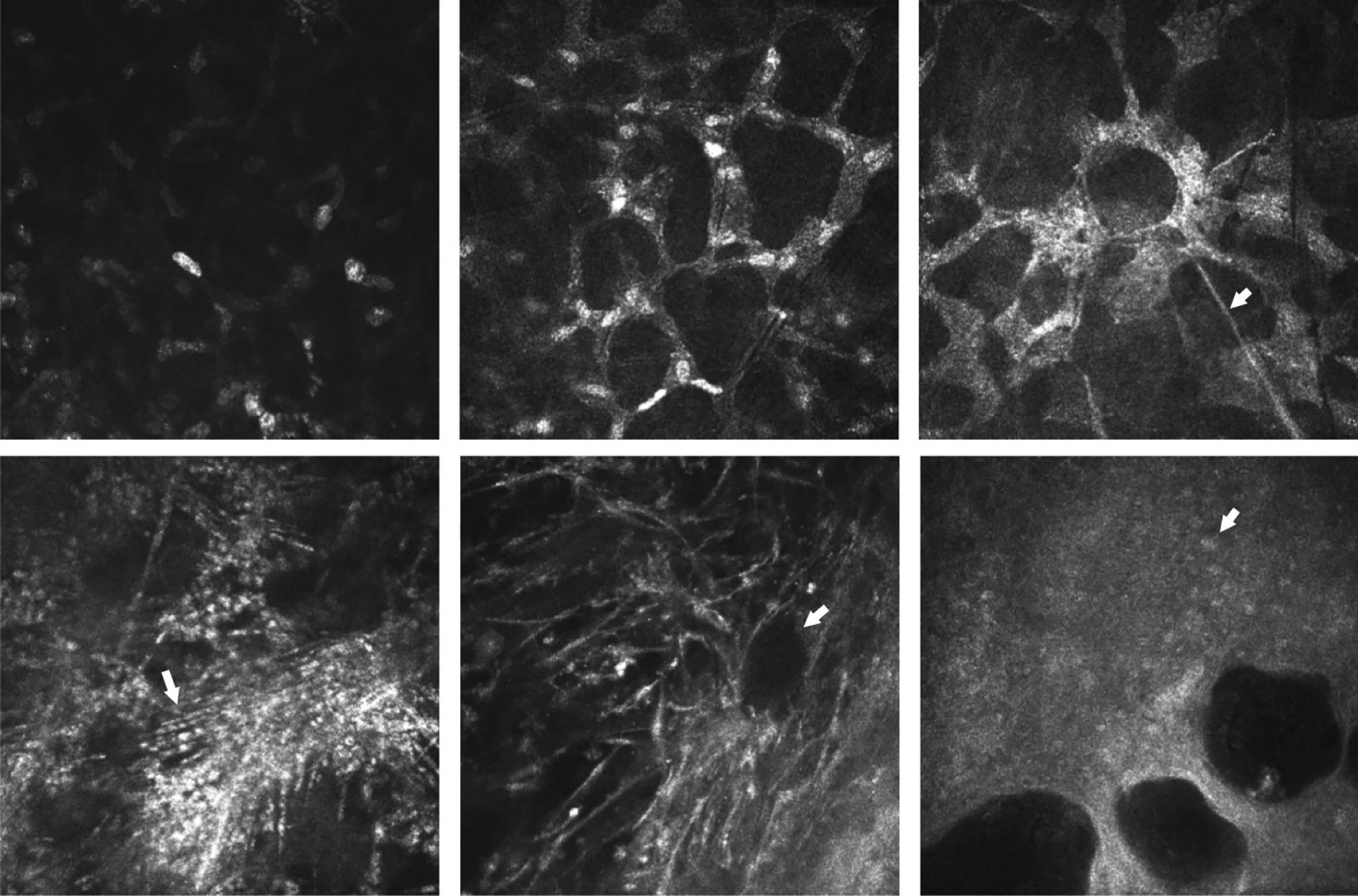
In vivo confocal microscopy images. (Top left) Normal keratocyte-like morphology (bright ovoid nuclei, barely visible cellular processes); (Top middle) “stellate intercellular connectivity with nuclei visible” (bright ovoid nuclei and broad bright cellular processes interconnected in a honeycomb network); (Top right) “stellate intercellular connectivity with lack of visible nuclei.” (Bottom left) Linear “spindles” (arrow); (Bottom middle) bullae in stroma (arrow); (Bottom right) bullae in epithelium (arrow showing epithelial cell nucleus).

**Figure 3:**
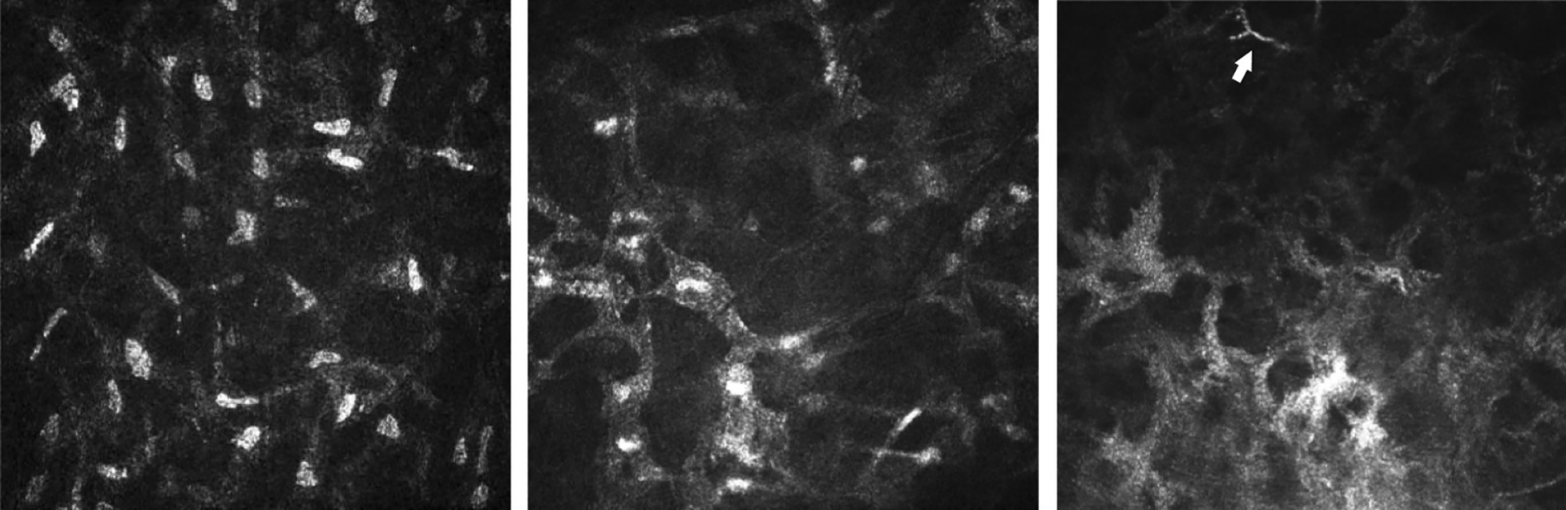
Additional in vivo confocal microscopy images show the normal keratocyte-like morphology in the anterior stroma (Left), stellate interconnected cellular processes with nuclei (Middle), and stellate interconnected without nuclei (Right; arrow highlights fungal filament).

**Figure 4:**
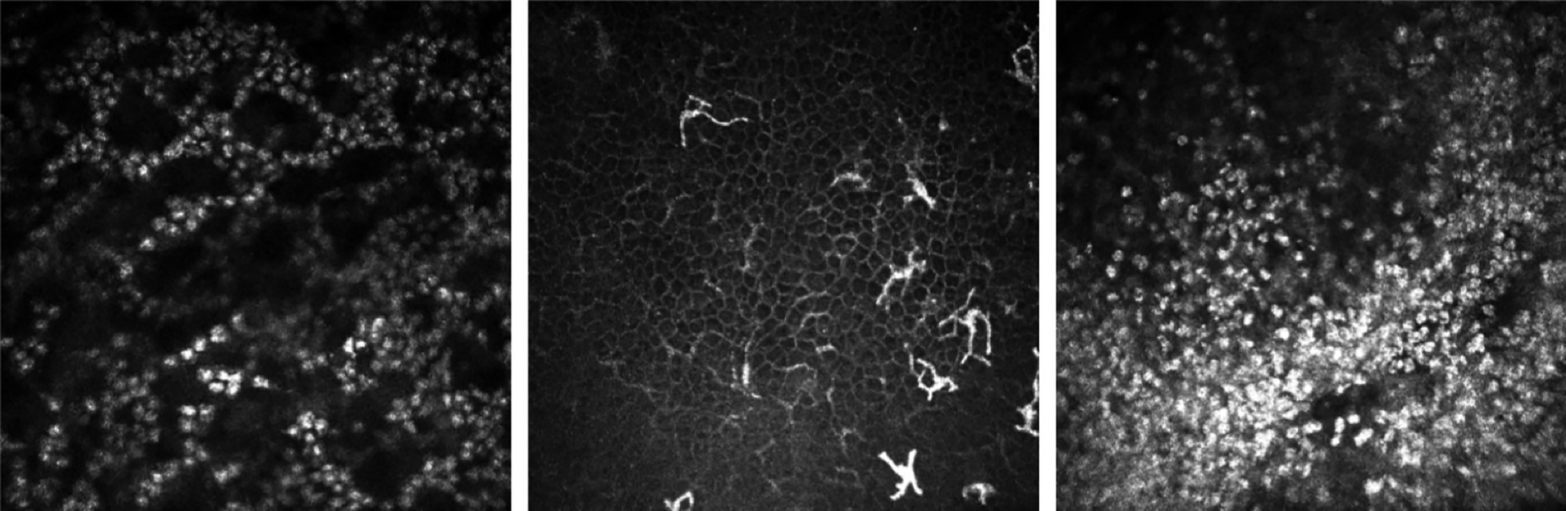
Appearance of inflammatory cell infiltrate in in vivo confocal microscopy images of microbial keratitis: (Left) inflammatory cells in a honeycomb distribution; (Middle) “dendritiform cells” in the basal epithelial layer; (Right) inflammatory cell infiltrate in a nonspecific distribution in the anterior stroma.

IVCM images were assigned a random identification number and were shuffled into a random order after removal of patient-identifying data. A single experienced grader performed all image grading and was masked to the clinical features and microbiological diagnosis. All data were recorded directly into a Microsoft Access database. The grading scheme included presence/absence of fungal hyphae, including presence/absence of spore-like structures (Figure 1), *Acanthamoeba* cyst features (double-wall, bright spot, signet ring, line, or cluster formation of cysts; Figure 1) or *Nocardia* sp. beaded filaments (Figure 1). Corneal stromal cellular appearances were graded as presence/absence of the following (as shown in Figures 2 and 3): “normal keratocyte-like morphology”—bright ovoid nuclei with barely visible processes (Figure 2, Top left; Figure 3, Left); “stellate cellular processes”—bright broad, bright interconnected cellular processes in a honeycomb formation either with bright ovoid nuclei (Figure 2, Top middle; Figure 3, Middle) or without nuclei (Figure 2, Top right; Figure 3, Right); “spindles”—linear bright nonbranching structures, often multiple and parallel to each other (Figure 2, Bottom left); and “granules”—small white opacities approximately 1-2 μm in diameter and present within the cells, either within the nuclear region or within the cellular processes (Figure 1, Middle). Other features included in the grading were presence/absence of “bullae” in the stroma or epithelium (Figure 2, Bottom right). The presence/absence of inflammatory cell appearances were also graded (Figure 4): “inflammatory cells in a honeycomb distribution”—bright round cells in alignment in a honeycomb contour (Figure 4, Left); “inflammatory cell infiltrate in a nonspecific distribution,” where a probable inflammatory cell infiltrate was detected but no honeycomb distribution was observed (Figure 4, Right), or “dendritiform cells,” either in the basal epithelial layer (basal DFCs; Figure 4, Middle) or in the stroma (stromal DFCs). Images from the anterior half of the cornea (0-250 μm as measured using the IVCM pachymeter) or the posterior half (>250 μm) were graded separately. Acellular regions with homogenous reflectivity were classified as “scar” tissue.

### Statistical Methods

All statistical analyses were performed in Stata 12.1 (StataCorp, College Station, Texas, USA). We used standardized ordinal grading scales (ie, cellular feature present/absent), as has been previously used in ophthalmic histopathology studies of microbial keratitis.^20^ Baseline demographic features and IVCM features were compared between BK, AK, FK, and microbiologically negative groups using χ^2^ test for proportions and Kruskal-Wallis test for continuous variables with nonparametric distribution. Logistic regression analysis was performed with the dependent variable as BK, AK, or FK vs all other ulcers combined, and with IVCM features as the independent variables. Initial analyses were performed with individual IVCM feature variables (adjusted for age, sex, ulcer size, and symptom duration), then the final multivariable analyses were also adjusted for any exposure (IVCM feature) where *P* < .1 in the initial analysis. Separate logistic regression analyses were performed for anterior and posterior corneal variables. Post hoc sub-group analysis was performed to compare IVCM features in *Aspergillus* keratitis to *Fusarium* keratitis to identify any differences in IVCM features between ulcers caused by these 2 fungi, which are the commonest causes of FK in the study cohort.^1^ All regression analyses were adjusted for age, sex, symptom duration, and ulcer stromal infiltrate size (defined as the geometric mean of longest stromal infiltrate diameter and its perpendicular diameter). Owing to collinearity, we only used ulcer stromal infiltrate size as a marker of disease severity, and did not include other signs (eg, ulcer depth, epithelial defect size).

## Results

Of the 239 participants enrolled in the study, 17 were excluded owing to being microbiologically negative (ie, no organism detected on culture, light microscopy, or IVCM) and 4 were excluded owing to mixed bacterial/fungal infection (culture-positive for bacteria and positive for fungal hyphae on light microscopy and/or IVCM). Of the remaining 218 participants, 183 were diagnosed with fungal keratitis, 18 with *Acanthamoeba* keratitis, and 17 with bacterial keratitis, as summarized in Table 1. The baseline demographic profile of participants within each group (BK, AK, FK) showed no statistically significant differences in age, sex, presenting visual acuity, or proportion of ulcers with deep involvement of the posterior cornea (Table 2). However, in the AK group the symptom duration (median 30 days, *P* < .001) and ulcer size (median 6.8 mm diameter, *P* < .001) were greater than for all other ulcers. A total of 3153 volume scans consisting of 126 120 images were obtained at the enrollment visit in all patients (median 12 volume scans per patient, interquartile range 9-16). We were able to perform IVCM imaging of the posterior half of the cornea in 57 ulcers (Table 3), the majority of which were fungal (81%, 46/57), and culture-positive for *Fusarium* sp. (n = 22).

**Table 1:** Causative Organisms Identified by Culture, Light Microscopy, and In Vivo Confocal Microscopy

	N	%
Fungi (n = 183)		
Culture-positive (n = 144):		
*Fusarium* sp.	73	33.5%
*Aspergillus* sp.	33	15.1%
Curvularia sp.	5	2.3%
Exserohilum sp.	4	1.8%
Lasiodiplodia sp.	2	0.9%
Cylindrocarpon sp.	1	0.4%
Bipolaris sp.	1	0.4%
Unidentified hyaline fungi	14	6.4%
Unidentified dematiaceous fungi	11	5.0%
Culture-negative but light microscopy–positive for fungi	30	13.8%
Culture-negative but IVCM-positive for fungi	9	4.1%
*Acanthamoeba* (n = 18)		
Culture-positive	17	7.8%
Culture-negative but IVCM-positive for *Acanthamoeba*	1	0.5%
Bacteria (n = 17)		
Culture-positive (n = 17):		
*Streptococcus pneumoniae*	9	4.1%
*Nocardia* sp.	3	1.4%
*Pseudomonas aeruginosa*	2	0.9%
*Aeromonas* sp.	1	0.4%
*Streptococcus viridans*	1	0.4%
*Staphylococcus epidermidis*	1	0.4%
Total	218	100%

IVCM = in vivo confocal microscopy.

**Table 2:** Baseline Characteristics of Study Participants

	Fungal Keratitis (77.4%, N = 183)	Acanthamoeba Keratitis (8.3%, N = 18)	Bacterial Keratitis (7.8%, N = 17)	*P* Value^b^
Median age, years (IQR)	50 (36-58)	39 (34-55)	60 (46-65)	.104
Male sex, n (%)	118 (64.8%)	11 (61.1%)	10 (58.8%)	.871
Symptom duration, median number of days (IQR)	7 (4-10)	30 (7-60)	8 (4-14)	<.001
Baseline visual acuity, median logMAR (IQR)	1.8 (0.6-1.8)	1.8 (1.7-1.8)	1.7 (1.7-1.8)	.244
Ulcer stromal infiltrate size,^a^ mm (median, IQR)	4.4 (3.3-5.5)	6.8 (5.3-8.0)	3.7 (3.2-5.0)	<.001
Deep infiltrate involving posterior one-third of cornea, n (%)	113 (62.1%)	13 (72.2%)	12 (70.6%)	.585

a Ulcer stromal infiltrate size calculated as geometric mean of longest diameter and perpendicular diameter.

b Differences between all 3 groups assessed for statistical significance using χ^2^ test for proportions (sex, ulcer depth) and Kruskal-Wallis test for continuous nonparametric variables.

**Table 3:** Cellular Features Detected Within In Vivo Confocal Microscopy Images of Bacterial, Fungal, *Acanthamoeba*, and Microbiologically Negative Keratitis

Corneal Location	IVCM Features	Fungal Keratitis (N = 183)	Acanthamoeba Keratitis (N = 18)	Bacterial Keratitis (N = 17)	*P* Value^a^
Anterior	Normal keratocyte-like morphology	141 (77%)	7 (39%)	14 (82%)	.001
	Stellate cellular processes with nuclei	119 (65%)	6 (33%)	11 (65%)	.029
	Stellate cellular processes no nuclei	58 (32%)	10 (56%)	3 (18%)	.047
	Spindles	132 (72%)	14 (78%)	13 (76%)	.826
	Granules	108 (59%)	13 (72%)	11 (65%)	.514
	Epithelial bullae	18 (10%)	0 (0%)	7 (41%)	<.001
	Stromal bullae	19 (10%)	1 (6%)	8 (47%)	<.001
	Inflammatory cells (honeycomb)	90 (49%)	1 (6%)	6 (35%)	.001
	Inflammatory cells (nonspecific)	42 (23%)	3 (17%)	6 (35%)	.403
	Basal DFCs	97 (53%)	7 (39%)	14 (82%)	.027
	Stromal DFCs	19 (10%)	5 (28%)	4 (23%)	.043
	Scar	19 (10%)	4 (22%)	3 (18%)	.251
	Fungal spore-like structures	6 (3%)	0 (0%)	0 (0%)	-

Corneal Location	IVCM Features	Fungal Keratitis (N = 46)	Acanthamoeba Keratitis (N = 7)	Bacterial Keratitis (N = 4)	*P* Value^a^
Posterior	Normal keratocyte-like morphology	31 (67%)	4 (57%)	4 (100%)	.320
	Stellate cellular processes with nuclei	35 (76%)	2 (29%)	3 (75%)	.037
	Stellate cellular processes no nuclei	10 (22%)	5 (71%)	1 (25%)	.024
	Spindles	30 (65%)	6 (86%)	2 (50%)	.430
	Granules	22 (48%)	6 (86%)	2 (50%)	.173
	Inflammatory cells (honeycomb)	11 (24%)	1 (14%)	0 (0%)	.476
	Inflammatory cells (nonspecific)	8 (17%)	0 (0%)	2 (50%)	.111
	Stromal DFCs	2 (4%)	0 (0%)	0 (0%)	.780
	Scar	0 (0%)	1 (14%)	0 (0%)	.026
	Fungal spore-like structures	2 (4%)	0 (0%)	0 (0%)	-

DFCs = dendritiform cells; IVCM = in vivo confocal microscopy.

a Statistical significance of difference between all 3 groups assessed using χ^2^ test.

### In Vivo Confocal Microscopy Cellular Changes In Fungal Keratitis

The IVCM feature that occurred most frequently in the anterior stroma in FK compared to all other ulcers was the presence of inflammatory cells in a honeycomb distribution, found in 49% of FK (90/183) compared to 20% of all nonfungal ulcers (7/35, *P* = .001, Table 3). In the logistic regression analysis, stromal bullae were independently associated with nonfungal rather than fungal ulcers (odds ratio [OR] 0.31, 95% confidence interval [CI] 0.11-0.82, *P* = .018, Table 4). A honeycomb distribution of inflammatory cells in the absence of stromal bullae was more strongly associated with FK (OR 3.31, 95% CI 1.02-10.77, *P* = .046) than in the presence of stromal bullae (OR 0.47, 95% CI: 0.15-1.45, *P* = .189). In the multivariable analysis the evidence of an association between fungal ulcers and inflammatory cells in a honeycomb distribution disappeared upon inclusion of the stromal bullae variable, and so the former was not included in the final multivariable model. In the posterior cornea, there were no specific features that were associated with FK.

**Table 4:** Univariable and Multivariable Odds Ratios for In Vivo Confocal Microscopy Features Associated With Bacterial, *Acanthamoeba*, and Fungal Keratitis

Pathogen	Corneal Location	Variable	Univariable OR (95% CI)	*P* Value	Multivariable OR (95% CI)	*P* Value
FK vs all others	Anterior	Stromal bullae	0.31 (0.11-0.82)	.018	0.31 (0.11-0.82)	.018
		Honeycomb inflammatory cell distribution	2.74 (1.01-7.40)	.047	-	-
		Honeycomb inflammatory cell distribution with no stromal bullae	3.31 (1.02-10.77)	.046		
		Honeycomb inflammatory cell distribution with stromal bullae present	0.47 (0.15-1.45)	.189		
	Posterior	-	-	-	-	-
*Aspergillus* sp. vs *Fusarium* sp.	Anterior	Stellate cellular processes with nuclei	0.23 (0.09-0.61)	.003	0.24 (0.09-0.65)	.005
		Stromal dendritiform cells	11.17 (1.65-75.44)	.013	11.05 (1.49-82.13)	.019
		Normal keratocytes	0.32 (0.11-0.93)	.036	-	-
		Granules	0.41 (0.16-1.02)	.055	-	-
		Anterior broken hyphae	2.46 (0.89-6.83)	.084	-	-
		Spindles	0.42 (0.15-1.17)	.097	-	-
	Posterior	-	-		-	-
AK vs all others	Anterior	Normal keratocyte-like morphology	0.21 (0.06-0.79)	.022	0.21 (0.06-0.79)	.022
	Posterior	Stellate cellular processes with nuclei	0.08 (0.01-1.13)	.062	0.03 (<0.01-1.09)	.056
		Granules	25.01 (0.73-855.51)	.075	49.57 (0.94-2604.52)	.053
Steroid use vs all others	Anterior	*Acanthamoeba* cysts in cluster formation	9.98 (1.02-97.96)	.048	9.98 (1.02-97.96)	.048
		*Acanthamoeba* cysts in line formation	2.43 (0.36-16.48)	.363	-	-
BK vs all others	Anterior	Stromal bullae	9.99 (3.11-32.06)	<.001	9.99 (3.11-32.06)	<.001
		Epithelial bullae	5.72 (1.73-18.94)	.004	-	-
		Basal epithelial dendritiform cells	3.74 (1.00-13.91)	.049	-	-
		Stromal dendritiform cells	3.51 (0.88-14.09)	.076	-	-
	Posterior	-	-	-	-	-

AK = *Acanthamoeba* keratitis; BK = bacterial keratitis; CI = confidence interval; FK = fungal keratitis; OR = odds ratio.

FK sub-group analysis for *Aspergillus* sp. vs *Fusarium* sp. also shown. All analyses were adjusted for age, sex, ulcer size, and symptom duration.

On comparison of *Aspergillus* keratitis (n = 33) with *Fusarium* keratitis (n = 73), ulcers with anterior stromal dendritiform cells had over 10 times the odds of being an *Aspergillus* ulcer than those without (multivariable OR 11.05, 95% CI: 1.49-82.13, *P* = .019). However those ulcers with a stellate cellular appearance with visible nuclei present were associated with having one quarter of the odds of being *Fusarium* ulcers compared to those without (multivariable OR 0.24, 95% CI: 0.09-0.65, *P* = .005). No posterior stromal features were associated with *Aspergillus* or *Fusarium* ulcers.

With regard to fungal features, we observed spore-like structures in the anterior stromal IVCM images of 6 of the 183 FK cases (3%; Figure 1). Three of these cases were culture-positive for dematiaceous fungi (*Curvularia* sp. n = 1, *Exserohilum* sp. n = 1, unidentified dematiaceous fungus n = 1), 1 was culture-positive for *Aspergillus flavus*, and the remainder were culture-negative but light microscopy–positive for fungal filaments (n = 2). The median symptom duration for these 6 cases was 10 days (interquartile range, [IQR] 7-15 days), and the median stromal infiltrate size was 3.9 mm in diameter (IQR 3.2-9.7 mm).

### *Acanthamoeba* Keratitis

*Acanthamoeba* ulcers were less likely to have a normal keratocyte-like morphology in the anterior stroma compared to all other causes of MK (multivariable OR 0.21, 95% CI: 0.06-0.79, *P* = .022; Table 4). In the posterior stroma multivariable analysis, there was a lower strength of association for either appearance of stellate cellular processes with nuclei in non-AK ulcers (OR 0.03, 95% CI: <0.01-1.09, *P* = .056) or presence of intracellular granules in AK ulcers (OR 49.57, 95% CI: 0.94-2604.52, *P* = .053; Table 4).

*Acanthamoeba* cysts were observed mainly as highly reflective bright spots (16/18, 89%) or with a double-walled morphology (15/18, 83%), rather than the signet ring appearance (3/18, 17%); in 14 patients, both bright spot and double-wall cyst morphologies were present in the IVCM images. The cysts appeared to group together into lines (7/18, 39%) or clusters (6/18, 33%), as shown in Figure 1. Specifically, prior steroid use was more strongly associated with the formation of clusters (OR 9.98, 95% CI 1.02-97.96, *P* = .048) rather than lines of cysts (OR 2.43, 95% CI: 0.36-16.48, *P* = .363; Table 4).

### Bacterial Keratitis

Epithelial and anterior stromal bullae were the main features that were associated with bacterial keratitis in the univariable analysis compared to all other causes of MK (OR 5.72, 95% CI: 1.73-18.94, *P* = .004, and OR 9.99, 95% CI: 3.11-32.06, *P* < .001, respectively; Table 4). Stromal bullae alone remained strongly associated with BK in multivariable analysis; the reduction in strength of evidence to support association between BK and epithelial bullae when included with stromal bullae in the multivariable model may be attributable to the independent association of epithelial bullae with bacterial ulcers and also with stromal bullae (ie, most likely along the causal pathway). DFCs in the basal epithelial layer (univariable OR 3.74, 95% CI: 1.00-13.91, *P* = .049) and in the anterior stroma (univariable OR 3.51, 95% CI: 0.88-14.09, *P* = .076) were very weakly associated with bacterial keratitis rather than any other cause of MK, but this did not reach statistical significance in the multivariable model.

There were 3 ulcers that were culture-positive for *Nocardia* sp. We only observed possible *Nocardia* filaments within the IVCM images of 1 of these 3 ulcers (Figure 1). The grader also recorded the presence of *Nocardia*-like fine, beaded filaments in 1 other ulcer, which was culture-positive for an unidentified dematiaceous fungus.

## Discussion

Here we have described the cellular changes that occur in the cornea in MK as observed with IVCM at first presentation. In FK, which formed the majority of cases in this study of large ulcers, the only IVCM feature weakly associated with this disease was the presence of an anterior stromal honeycomb distribution of inflammatory cells. This specific honeycomb pattern of inflammatory cells is similar to that observed after abrasion injury in real-time in vivo HRT3 IVCM imaging of the mouse cornea; these inflammatory cells were identified as neutrophils using immunohistochemistry in the same tissue ex vivo, and their close interaction with keratocytes was found to be mediated through action of cell adhesion molecules.^9^ However, to our knowledge, this honeycomb distribution of migrating inflammatory cells has not been formally investigated in FK before. Neutrophils are recruited to the cornea very soon after the onset of infection in MK, even within hours, and this is mediated through release of chemokines in the cornea by host cells (eg, CXCL1, CXCL5, IL8).^14^, ^21^

The nature of the corneal cellular response to fungal infection may also differ in *Aspergillus* vs *Fusarium* keratitis; in the IVCM images of the anterior corneal stroma, we observed associations between dendritiform cells and *Aspergillus* ulcers in our subgroup analysis. Since *Aspergillus* keratitis is often more difficult to treat, with greater risk of poor outcomes, larger studies are needed to more fully ascertain whether there may be IVCM cellular features that are associated with this fungus to aid diagnosis and management of these cases.^22^

In 6 of the FK cases, we detected fungal spore-like structures that were present along hyphae in the anterior stroma. These most likely represent chlamydospores, which are thick-walled structures along hyphae that typically occur in fungi that have depleted their local nutrient supply.^23^ Chlamydospores have been previously reported in corneal scrapings from human FK, predominantly in ulcers that were culture-positive for dematiaceous fungi such as *Curvularia* sp.^17^ In our study, 3 of the 6 ulcers with spore-like structures detected on IVCM were culture-positive for dematiaceous fungi. Others have shown that the presence of fungal spores within tissues is frequently associated with disseminated disease and poor prognosis.^24^ Similarly in FK, the appearance of spore-like structures within IVCM images may be an indicator of worsening of disease, and so further studies are required to elucidate its prognostic value.

In AK, the main IVCM features associated were a lack of normal keratocyte-like morphology in the anterior stroma compared to the other causes of MK. *Acanthamoeba* are able to kill keratocytes through other mechanisms such as direct cytopathic effects, phagocytosis, and induction of apoptosis or necrosis, as shown in both in vitro studies and histologic studies, although apoptosis is most likely to be the predominant method by which keratocyte death occurs in AK.^25^, ^26^ Although we were not able to perform immunohistochemical studies to confirm apoptosis, others have done so and found through the use of TUNEL staining that apoptosis of keratocytes does indeed occur throughout the corneal stroma in human AK, BK, and FK, and particularly in the posterior stroma in AK.^20^, ^26^ We were only able to study a small number of *Acanthamoeba* ulcers in this study, and so larger studies are required to confirm these IVCM findings.

We also found that AK cyst-like structures formed clusters, particularly in ulcers that had undergone treatment with topical steroid prior to presentation. Yokogawa and associates observed that clusters of *Acanthamoeba* cysts in the Bowman membrane were present in cases of persistent AK, and that a high proportion of these patients had used topical steroid therapy prior to presentation.^27^ Zhang and associates also noted that the formation of lines or clusters of AK cysts was associated with poor prognosis in their series of 29 patients with AK, although steroid use was not mentioned.^28^ Reasons for in vivo cluster formation of *Acanthamoeba* cysts with or without steroid exposure remain to be elucidated, but prior studies have shown that *Acanthamoeba*e are able to adhere to multiple surfaces, including contact lenses,^29^ corneal extracellular matrix components (eg, collagens and laminins),^30^ and host corneal epithelial cells.^31^ Exposure of *Acanthamoeba* cysts to dexamethasone increases their cytopathic effect on host corneal cells, and this could be one reason for poor prognosis in patients treated with topical steroid alone.^32^ Larger studies are needed to identify whether the formation of clusters of *Acanthamoeba* cysts in IVCM images is a useful prognostic indicator in AK.

DFCs in the IVCM images of ocular disease have been used as a predictor of causative organism. Cruzat and associates studied the presence of basal DFCs only (not stromal DFCs) in HRT3 IVCM images of AK, BK and FK, and found that AK had a higher density of DFCs in this region of the cornea.^7^ We found that a higher proportion of IVCM images from the BK group had basal DFCs, rather than in AK or FK. The difference may be related to prior steroid use, since many of the BK patients in the study by Cruzat and associates had used topical steroids beforehand, whereas only 1 BK patient in our study had a history of steroid use. Multiple cell types can take on a dendritiform morphology, as observed with confocal microscopy (both IVCM and ex vivo). Corneal tissue–resident macrophages, dendritic cells, and even keratocytes can possess this elongated, dendritiform cell shape, in addition to bone marrow–derived myeloid cells that have migrated into the inflamed cornea.^6^, ^33^, ^34^, ^35^ Future studies directly comparing IVCM imaging with immunostaining of the same tissue ex vivo would aid in identifying the cell of origin of the morphologies that we have described in this report, and would provide further information on the pathogenesis of disease.

In contrast to AK and FK, the occurrence of bullae in the epithelium and stroma were associated with BK. Epithelial bullae have been observed in IVCM images of Fuchs endothelial dystrophy in the past, and ascribed to tissue edema causing microcysts within the epithelial layer.^36^ The larger bullae seen within the corneal stroma may be an indication of stromal tissue damage. Most of the BK cases in this study were culture-positive for *Streptococcus pneumoniae*. This organism contributes to host tissue damage through multiple mechanisms, including release of reactive oxygen species^37^ and excessive stimulation of host cells (eg, neutrophils) to release matrix metalloproteinases that can also destroy host tissue.^38^ Control of this damage through use of topical steroid treatment early on in bacterial corneal ulceration may have some impact on improving final visual outcome in large ulcers that are in the visual axis.^39^ IVCM may be a useful tool for monitoring the effect of any treatment regime on the stromal necrotic response.

*Nocardia* sp. have been documented to appear as thin beaded filamentous structures in IVCM images^18^, ^19^ and are therefore one of the few bacterial causes of keratitis that can be visualized with this imaging modality.^15^ *Nocardia* sp. filaments are smaller in diameter than *Aspergillus* or *Fusarium* fungal hyphae (up to 1 μm compared to 3-6 μm for filamentous fungi).^13^, ^40^ We only observed thin beaded filaments in the IVCM images of 1 out of the 3 *Nocardia* ulcers in our study, and also in 1 ulcer that was culture-positive for a dematiaceous fungus. As such, it may not always be possible to rely on direct visualization of thin beaded filaments in IVCM images of MK to make the diagnosis of *Nocardia* keratitis.

A limitation of our study is that we were able to enroll only a small number of bacterial and *Acanthamoeba* ulcers. Since this was a prospective cohort study, the observation that the majority of our cases were fungal reflects the distribution of causative organisms of large ulcers in South India. Larger studies are needed in the future to more fully elucidate the IVCM features that we have reported for bacterial and *Acanthamoeba* keratitis. Also, we chose to only enroll large ulcers, as we felt that these can often pose a greater diagnostic challenge and frequently have a worse visual outcome. Ulcers of a smaller size at presentation may have lesser tissue damage at presentation, and so different IVCM cellular findings, which need to be investigated in the future.

In summary, here we show that patterns of cellular changes as detected with IVCM may be helpful in predicting the causative organism in MK. In addition to diagnosing the pathogen, IVCM allows an insight into the histology of the living cornea during infection and the cellular host response. Future studies are required to explore the use of IVCM in particular for monitoring therapeutic response.

## Figures and Tables

**Figure:**
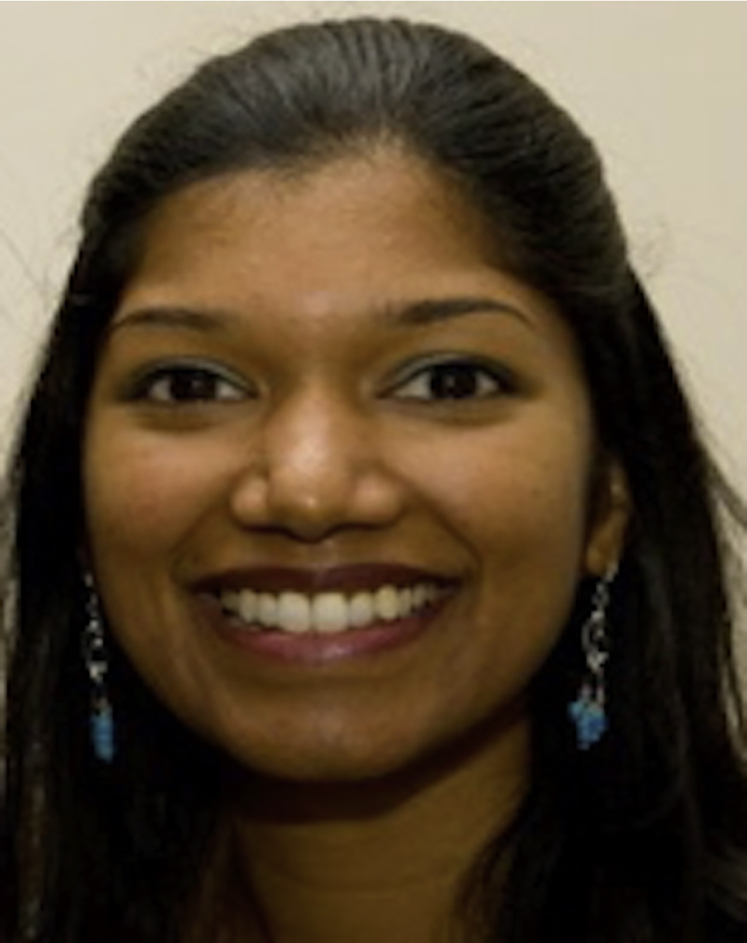
Dr. Jaya Chidambaram, BSc(Hons), MBBS, MRCOphth, completed her ophthalmology training in London, UK, and a research fellowship on trachoma and microbial keratitis at the Proctor Foundation, University of California San Francisco. She is currently pursuing a Wellcome Trust PhD on microbial keratitis at the London School of Hygiene and Tropical Medicine. Her PhD research, based in Aravind Eye Hospital, India, has won awards at the UK Royal College of Ophthalmologists' Annual Congress and Asia ARVO.

## Supplemental Data

Data Profile

## References

[1] Chidambaram J.D., Prajna N.V., Larke N.L. (2016). Prospective study of the diagnostic accuracy of the in vivo laser scanning confocal microscope for severe microbial keratitis. Ophthalmology.

[2] Hovakimyan M., Falke K., Stahnke T. (2014). Morphological analysis of quiescent and activated keratocytes: a review of ex vivo and in vivo findings. Curr Eye Res.

[3] Jester J.V., Barry-Lane P.A., Cavanagh H.D., Petroll W.M. (1996). Induction of alpha-smooth muscle actin expression and myofibroblast transformation in cultured corneal keratocytes. Cornea.

[4] Jester J.V., Brown D., Pappa A., Vasiliou V. (2012). Myofibroblast differentiation modulates keratocyte crystallin protein expression, concentration, and cellular light scattering. Invest Ophthalmol Vis Sci.

[5] Cavanagh H.D., Petroll W.M., Alizadeh H., He Y.G., McCulley J.P., Jester J.V. (1993). Clinical and diagnostic use of in vivo confocal microscopy in patients with corneal disease. Ophthalmology.

[6] Jester J.V., Huang J., Petroll W.M., Cavanagh H.D. (2002). TGFbeta induced myofibroblast differentiation of rabbit keratocytes requires synergistic TGFbeta, PDGF and integrin signaling. Exp Eye Res.

[7] Cruzat A., Witkin D., Baniasadi N. (2011). Inflammation and the nervous system: the connection in the cornea in patients with infectious keratitis. Invest Ophthalmol Vis Sci.

[8] Shi W., Li S., Liu M., Jin H., Xie L. (2008). Antifungal chemotherapy for fungal keratitis guided by in vivo confocal microscopy. Graefes Arch Clin Exp Ophthalmol.

[9] Hanlon S.D., Smith C.W., Sauter M.N., Burns A.R. (2014). Integrin-dependent neutrophil migration in the injured mouse cornea. Exp Eye Res.

[10] Hau S.C., Dart J.K., Vesaluoma M. (2010). Diagnostic accuracy of microbial keratitis with in vivo scanning laser confocal microscopy. Br J Ophthalmol.

[11] Kanavi M.R., Javadi M., Yazdani S., Mirdehghanm S. (2007). Sensitivity and specificity of confocal scan in the diagnosis of infectious keratitis. Cornea.

[12] Vaddavalli P.K., Garg P., Sharma S., Sangwan V.S., Rao G.N., Thomas R. (2011). Role of confocal microscopy in the diagnosis of fungal and acanthamoeba keratitis. Ophthalmology.

[13] Winchester K., Mathers W.D., Sutphin J.E. (1997). Diagnosis of Aspergillus keratitis in vivo with confocal microscopy. Cornea.

[14] Chidambaram J.D., Kannambath S., Srikanthi P. (2017). Persistence of innate immune pathways in late stage human bacterial and fungal keratitis: results from a comparative transcriptome analysis. Front Cell Infect Microbiol.

[15] Labbe A., Khammari C., Dupas B. (2009). Contribution of in vivo confocal microscopy to the diagnosis and management of infectious keratitis. Ocul Surf.

[16] Fust A., Toth J., Simon G., Imre L., Nagy Z.Z. (2017). Specificity of in vivo confocal cornea microscopy in Acanthamoeba keratitis. Eur J Ophthalmol.

[17] Gajjar D.U., Pal A.K., Ghodadra B.K., Vasavada A.R. (2013). Microscopic evaluation, molecular identification, antifungal susceptibility, and clinical outcomes in fusarium, Aspergillus, and dematiaceous keratitis. BioMed Res Int.

[18] Johansson B., Fagerholm P., Petranyi G., Claesson Armitage M., Lagali N. (2017). Diagnostic and therapeutic challenges in a case of amikacin-resistant Nocardia keratitis. Acta Ophthalmol.

[19] Vaddavalli P.K., Garg P., Sharma S., Thomas R., Rao G.N. (2006). Confocal microscopy for Nocardia keratitis. Ophthalmology.

[20] Vemuganti G.K., Reddy K., Iftekhar G., Garg P., Sharma S. (2004). Keratocyte loss in corneal infection through apoptosis: a histologic study of 59 cases. BMC Ophthalmol.

[21] Lin M., Carlson E., Diaconu E., Pearlman E. (2007). CXCL1/KC and CXCL5/LIX are selectively produced by corneal fibroblasts and mediate neutrophil infiltration to the corneal stroma in LPS keratitis. J Leukoc Biol.

[22] Lalitha P., Prajna N.V., Kabra A., Mahadevan K., Srinivasan M. (2006). Risk factors for treatment outcome in fungal keratitis. Ophthalmology.

[23] Schippers B., Old K.M. (1974). Factors affecting chlamydospore formation by Fusarium solani f. cucurbitae in pure culture. Soil Biol Biochem.

[24] Liu K., Howell D.N., Perfect J.R., Schell W.A. (1998). Morphologic criteria for the preliminary identification of Fusarium, Paecilomyces, and Acremonium species by histopathology. Am J Clin Pathol.

[25] Takaoka-Sugihara N., Yamagami S., Yokoo S., Matsubara M., Yagita K. (2012). Cytopathic effect of Acanthamoeba on human corneal fibroblasts. Mol Vis.

[26] Vemuganti G.K., Sharma S., Athmanathan S., Garg P. (2000). Keratocyte loss in Acanthamoeba keratitis: phagocytosis, necrosis or apoptosis?. Indian J Ophthalmol.

[27] Yokogawa H., Kobayashi A., Yamazaki N. (2012). Bowman’s layer encystment in cases of persistent Acanthamoeba keratitis. Clin Ophthalmol.

[28] Zhang X., Sun X., Jiang C. (2014). A new in vivo confocal microscopy prognostic factor in Acanthamoeba keratitis. J Fr Ophtalmol.

[29] Kilvington S. (1993). Acanthamoeba trophozoite and cyst adherence to four types of soft contact lens and removal by cleaning agents. Eye (Lond).

[30] Rocha-Azevedo B.D., Jamerson M., Cabral G.A., Silva-Filho F.C., Marciano-Cabral F. (2009). Acanthamoeba interaction with extracellular matrix glycoproteins: biological and biochemical characterization and role in cytotoxicity and invasiveness. J Eukaryot Microbiol.

[31] Panjwani N. (2010). Pathogenesis of acanthamoeba keratitis. Ocul Surf.

[32] McClellan K., Howard K., Niederkorn J.Y., Alizadeh H. (2001). Effect of steroids on Acanthamoeba cysts and trophozoites. Invest Ophthalmol Vis Sci.

[33] Peebo B.B., Fagerholm P., Traneus-Rockert C., Lagali N. (2011). Cellular level characterization of capillary regression in inflammatory angiogenesis using an in vivo corneal model. Angiogenesis.

[34] Hamrah P., Zhang Q., Liu Y., Dana M.R. (2002). Novel characterization of MHC class II-negative population of resident corneal Langerhans cell-type dendritic cells. Invest Ophthalmol Vis Sci.

[35] Hamrah P., Liu Y., Zhang Q., Dana M.R. (2003). Alterations in corneal stromal dendritic cell phenotype and distribution in inflammation. Arch Ophthalmol.

[36] Alomar T.S., Al-Aqaba M., Gray T., Lowe J., Dua H.S. (2011). Histological and confocal microscopy changes in chronic corneal edema: implications for endothelial transplantation. Invest Ophthalmol Vis Sci.

[37] Rai P., Parrish M., Tay I.J. (2015). Streptococcus pneumoniae secretes hydrogen peroxide leading to DNA damage and apoptosis in lung cells. Proc Natl Acad Sci U S A.

[38] Vissers M., Hartman Y., Groh L., de Jong D.J., de Jonge M.I., Ferwerda G. (2014). Recognition of Streptococcus pneumoniae and muramyl dipeptide by NOD2 results in potent induction of MMP-9, which can be controlled by lipopolysaccharide stimulation. Infect Immun.

[39] Srinivasan M., Mascarenhas J., Rajaraman R. (2012). Corticosteroids for bacterial keratitis: the Steroids for Corneal Ulcers Trial (SCUT). Arch Ophthalmol.

[40] Brasnu E., Bourcier T., Dupas B. (2007). In vivo confocal microscopy in fungal keratitis. Br J Ophthalmol.

